# Monitoring Immobilized Elderly Patients Using a Public Provider Online System for Pressure Ulcer Information and Registration (SIRUPP): Protocol for a Health Care Impact Study

**DOI:** 10.2196/13701

**Published:** 2019-08-12

**Authors:** Eugenio Vera-Salmerón, Claudia Rutherford, Carmen Dominguez-Nogueira, María Pilar Tudela-Vázquez, Victor J Costela-Ruiz, Basilio Gómez-Pozo

**Affiliations:** 1 Distrito Sanitario Granada-Metropolitano (Servicio Andaluz de Salud) Armilla Spain; 2 Instituto de Investigación Biosanitaria de Granada (ibs.GRANADA) Granada Spain; 3 Unidades Asistenciales Churriana de la Vega y Peligros Granada Spain; 4 Beckett Senior Research Fellow Quality of Life Office University of Sydney Sydney Australia; 5 Departamento de Enfermería Facultad de Ciencias de la Salud Universidad de Granada Granada Spain; 6 Unidad Interniveles de Prevención Promoción y Vigilancia de la Salud Granada Spain

**Keywords:** primary health care, pressure ulcers, wound healing, health-related quality of life

## Abstract

**Background:**

Pressure ulcers represent a major challenge to patient safety in the health care context, presenting high incidence (from 7% to 14% in Spain) and increased financial costs (€400-600 million/year) in medical treatment. Moreover, they are a significant predictor of mortality. The prevention of pressure ulcers in long-term care centers and patients’ own homes is proposed as a priority indicator of health care quality. Early stage risk assessment and database recording are both crucial aspects of prevention, classification, diagnosis, and treatment.

**Objective:**

This project proposes a 3-year study of immobilized patients residing in the Granada-Metropolitan Primary Healthcare District (DSGM) and monitored via the Pressure Ulcer Information and Registration System (SIRUPP, Spanish initials). The project aims to estimate the incidence of PUs among immobilized elderly patients, analyze the health-related quality of life of these patients by using the Pressure Ulcer Quality of Life (PU-QoL) instrument in a sample of 250 patients, determine the average time to complete wound healing, estimate the rate of pressure ulcers–associated mortality, and assess the predictive value of the Braden and Mini Nutritional Assessment risk measurement scales in a sample of 1700 patients.

**Methods:**

The DSGM runs SIRUPP, which is linked to patients’ electronic health records. Currently, 17,104 immobilized patients are monitored under this system. Health-related quality of life will be measured by patient self-reports using the Spanish Pressure Ulcer Quality of Life questionnaire, following cross-cultural adaptation and psychometric validation with respect to the English-language version.

**Results:**

The project commenced in June 2017 and is expected to conclude in April 2020.

**Conclusions:**

This study addresses two main health outcomes—the time needed for wound healing and the mortality associated with pressure ulcers—both of which might be accounted for by variations in clinical practice and the health-related quality of life of patients with pressure ulcers.

**International Registered Report Identifier (IRRID):**

DERR1-10.2196/13701

## Introduction

### Background

Pressure ulcers (PUs) are injuries located in or under the skin, usually on a bony protrusion, resulting from pressure or shear forces [1,2]. They affect persons with reduced mobility and are associated with diverse health conditions and clinical settings. PUs may affect hospitalized patients, residents of nursing homes, the elderly, or persons living in their own homes, among others [3-7].

PUs represent a major public health problem, causing serious medical complications, severely reducing patients’ health-related quality of life (HRQoL), and greatly increasing the financial costs of medical treatment [8]. Studies have reported a three-fold increase in mortality among hospitalized patients and an increase of 6.4 days in the length of hospital stay among patients with PUs. Consequently, the prevention of PUs in long-term care centers is considered a priority indicator of health care quality [9,10].

Most PUs are considered avoidable if suitable preventative measures are taken [11]. The three most important risk factors for PUs are mobility-activity, perfusion, and skin state [12]. Other risk factors include advanced age, long stay in care centers, medical history of PUs, diabetes, low blood pressure, sensory neuropathy, falls, kidney and peripheral vascular diseases, and nutritional status [7-9,11-13].

Estimates of the incidence of PUs worldwide have been determined. In Belo Horizonte (Brazil), an incidence of 5.7% was reported for a cohort of 442 hospitalized patients [14], while in Italy, a multicenter cohort study of 1083 hospitalized elderly patients reported an incidence of 22.7% [15].

The latest survey in 2013 by the Spanish Advisory Panel on Pressure Ulcers and Chronic Wounds reported that the prevalence of PUs varied from 7% to 8.5% in hospitals, 12% to 14% in nursing homes, and 8% to 9% among patients living at home; 65.6% of PUs were diagnosed during hospital stays or in nursing homes and 29.6% were diagnosed in home-care patients [16]. In the Granada-Metropolitan Primary Healthcare District (DSGM), which includes both urban and rural areas, the total immobilized patient population susceptible to the development of PUs is 16,467 (15% of persons aged≥65 years) [17]. According to the findings of the 4th National Study on PUs, for this population, the estimated prevalence of PUs would be 8%-9%, or 1300-1500 patients [16]. In total, the DSGM has 72 nursing homes for the elderly, with 4700 residents aged≥65 years. To record the incidence of PUs, the DSGM designed a specific instrument—the Pressure Ulcer Information and Registration System (SIRUPP, Spanish initials)—which has been integrated into patient electronic health records at the DSGM. SIRUPP has been presented, as a good practice initiative, to the EU Network on Patient Safety and Quality of Care [18]. It has been piloted in the DSGM and used as an instrument for clinical follow-up. The large number of immobilized patients currently registered in SIRUPP is expected to facilitate the follow-up stage of this research study.

The most commonly used tools to evaluate PU risk are the Braden scale and the Norton scale, although the latter does not consider the abovementioned risk factors. Accordingly, most research attention has been paid to the Braden scale and its psychometric properties [2,11,19]. Many factors representing the risk of PU development are omitted from this scale. Therefore, and in view of the known relationship between patient age and comorbidity, the Braden scale is not considered to offer high predictive performance for patients aged over 80 years [13]. For this reason, the parallel implementation of specific tests to evaluate the nutrition levels of these older patients has been recommended. Research evidence supports the use of the Mini Nutritional Assessment scale (MNA) to evaluate PU risk as a complement to the Braden scale. The MNA scale is a useful means of evaluating the nutritional status of elderly persons [9,20-24]. The MNA - Short Form questionnaire (MNA-SF) takes into account the subject’s body mass index or the calf circumference, which is, perhaps, a more useful indicator for immobilized patients. For persons with cognitive impairment, the MNA test can be applied via an interviewer or relatives [25].

PUs can negatively impact the HRQoL [26]. A 1-year follow-up cohort study in Catalonia analyzed the factors associated with mortality and HRQoL in a sample of 1000 immobilized patients (mean age: 84 years), which included patients in a home-based health care program [27]. The study reported that comorbidity of PUs (measured by the Charlson index) was associated with a 14% higher risk of death. Stage I/II and III/IV PUs increased this risk by three and four times, respectively. When hospitalization exceeded 24 hours, mortality was 17% higher. On the contrary, a high self-perceived HRQoL (measured by the SF-12 health status questionnaire) and a low cognitive impairment were both associated with longer survival [28].

Patient-reported outcomes are of crucial importance in health care decision making [26,28]. To our knowledge, only one instrument has been specifically developed for patients with PUs, namely, the PU-QoL questionnaire [29,30].

From our literature review, we hypothesize that there is geographical heterogeneity in the incidence of PUs in the elderly. In addition, a high variability in clinical practice is expected, which will be expressed as heterogeneity in the time needed for the wounds to heal as well as differences in the mortality rates by place of residence. Furthermore, given the psychometric properties exhibited for some widely used questionnaires, it is expected that the MNA questionnaire on its own or in combination with the Braden scale will outperform the Braden scale as a predictive tool for the risk of PUs. Finally, we expect that the validation of the PU-QoL questionnaire in Spanish will preserve the psychometric properties of the original English version and allow for valid and precise estimation of the quality of life in patients with PUs.

### Objectives

The objectives of this study were as follows:

1. To estimate the incidence of PUs in immobilized elderly patients:
  - To estimate the time to full wound healing
  - To estimate the mortality associated with the presence of PUs
  - To compare the responsiveness to change and the predictive value of the Braden and MNA questionnaires
2. To analyze the HRQoL of immobilized elderly persons with PUs
3. To translate, adapt, and validate the PU-QoL questionnaire in Spanish
4. To estimate patients’ nutritional status (MNA-SF), cognitive status (the Reisberg Global Deterioration Scale) and functional independence (the Barthel scale)
5. To determine the PU-QoL questionnaire’s ability to differentiate between clinical groups (eg, PU severity) and its responsiveness to change (eg, wound healing)

## Methods

### Design

This study is based on SIRUPP, which is a part of the patient electronic health record system in Andalucía. The system was designed by the SIRUPP study coordinator in 2015 to provide professionals with a standardized registration system for PUs and was initiated in 2017 as part of a proposed 3-year follow-up of immobilized patients, with no PUs at the outset. The SIRUPP system has been presented as a good clinical practice initiative to the EU Patient Safety and Quality of Care project. Among other aims, SIRUPP is intended to facilitate the evaluation of variations in clinical practice and participants’ HRQoL. The incidence of PUs and the corresponding health outcomes will be assessed during the study, to be coordinated by the DSGM, located in the province of Granada (Spain). In Spain, primary health care districts are the main organizational public health structure for the planning, administration, and operational management of primary health services. Each district is structured into several primary health care zones (ZBS), each containing one or more health care centers. The DSGM contains 36 ZBSs and serves a population of 697,000 people. To achieve objectives 1 (incidence), 1-a (time elapsed until full wound healing), and 1-b (mortality), a pilot cohort study will be performed to monitor all immobilized patients registered in SIRUPP, presenting no PUs at the outset. The follow-up process will continue until the wound heals, the patient dies, or the project ends, whichever occurs first. To achieve objective 1-c (Braden and MNA questionnaires), we will conduct a repeated-measures prospective cohort study in a sample of immobilized patients without PUs registered in SIRUPP. With respect to HRQoL, objectives 2 and 3 will be addressed by means of a cross-sectional study in a sample of immobilized patients registered in SIRUPP. At this point of the project, patients who developed PUs since the start of the study will be recruited. Regarding objectives 4 (nutrition, cognition, and functional independence) and 5 (PU-QoL questionnaire), a repeated-measures cohort study (Tables 1 and 2) will be implemented for all immobilized patients registered in SIRUPP, who have developed PUs since the start of the study period.

Patients enter the SIRUPP registry as they fulfill the criteria established by the service portfolio in the Andalusian Health Service. Consequently, anybody who is at risk of impairment in their ability for moving, but not necessarily mobility-impaired at that time, is registered as *immobilized*. Therefore, the state of immobilization will be further categorized for all patients registered in SIRUPP by using the Braden mobility and activity subscales as well as the MNA-SF mobility subscale. This will provide additional up-to-date information on the actual state of patients’ mobility. To minimize the underreporting of immobilized patients, periodic monitoring of compliance with management agreements at all health centers within the DSGM will be audited. Furthermore, variations in clinical practice may lead to a classification bias of PUs against other types of chronic wounds. To forestall this possibility, the project will include training in this respect for all nurses responsible for the care of immobilized patients. In addition, SIRUPP software incorporates a protocol for the evaluation and staging of chronic wounds that ensures homogeneous, standardized categorization in clinical practice. Finally, a fieldwork coordinator in charge of all follow-up activities will ensure that all the information obtained is up to date.

**Table 1:** SIRUPP Project CADENCE for immobilized patients without PUs.

Periodicity schedule			Day 0	Day 7	Day 14	Day 21	Day 28	After 90 days
**Living at home**								
	**Braden**							
		None/low risk	✓				✓	✓
		Moderate/high risk	✓		✓		✓	✓
	MNA-SF^a^		✓		✓		✓	✓
**Living at nursing home**								
	Braden		✓	✓	✓	✓	✓	✓
	MNA-SF		✓	✓	✓	✓	✓	✓

^a^MNA-SF: Mini Nutritional Assessment - Short Form.

**Table 2:** SIRUPP Project CADENCE for immobilized patients with PUs.

Periodicity schedule			Day 0	Mo 1	Mo 3	Mo 6	Mo 9	Mo 12
**Living at home**								
	**MNA-SF^a^, score**							
		12-14	✓					✓
		8-11	✓		✓			
		0-7	✓	✓				
	Barthel		✓			✓		
	GDS^b^		✓			✓		
	PU-QoL^c^		✓			✓		
**Living at nursing home**								
	**MNA-SF, score**							
		12-14	✓		✓			
		8-14	✓	✓				
		0-7	✓			✓		
	GDS		✓			✓		
	PU-QoL		✓				✓	

^a^MNA-SF: Mini Nutritional Assessment – Short Form.

^b^GDS: Global Deterioration Scale.

^c^PU-QoL: Pressure Ulcer Quality of Life Questionnaire.

### Participants

The inclusion criteria for patients are immobilized status, male or female gender, age≥65 years, living in their own home or a nursing home for the elderly, and receiving treatment at primary health care centers in the DSGM. Patients who understand the purpose of the study can provide informed consent to participate on their own. For patients with cognitive impairment, their legal guardians can understand the purpose of the study and provide informed consent.

Exclusion criteria are as follows: refusal to participate, patients with cognitive impairment that prevents them from understanding the purpose of the study, patients who do not grant informed consent, patients with cognitive impairment whose legal guardians refuse consent to participate, and terminally ill patients.

### Data Management

**Figure 1:**
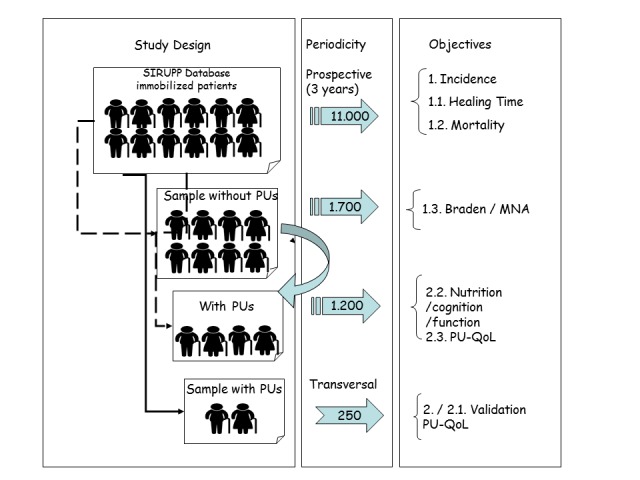
SIRUPP (Pressure Ulcer Information and Registration System) sample size and study design. PU: pressure ulcer; PU-QoL: Pressure Ulcer Quality of Life; MNA: Mini Nutritional Assessment.

With regard to the sample size, for objectives 1 (incidence) and 1-a (time elapsed until full wound healing) and 1-b (mortality), all immobilized patients registered in SIRUPP will be followed up. To estimate the Braden and MNA predictive values (objective 1-c), we calculated that a sample size of 1700 immobilized patients will be required. Test sensitivity and specificity values of 65% and 70%, respectively, are assumed, together with a PU prevalence of 5%, a statistical confidence level of 95%, and an absolute precision of 10%. For objective 3 (validation of the PU-QoL questionnaire), a sample of 250 patients with PUs will be analyzed (Figure 1). Table 3 presents the variables on sociodemographic and health characteristics.

### Data Analysis

Data analysis will be performed as follows:

1. To determine PU incidence, the rate will be calculated between the number of new cases of PU over the summed person-years of observation during the follow-up period.
  - To estimate time until wound-healing, survival analysis fitting a Weibull model will be used.
  - To estimate mortality associated with PUs, the mortality rate will be calculated as the number of deaths occurring during the follow-up period over the size of the population participating in the study.
  - To assess the responsiveness to change of the Braden and MNA questionnaires, multiple regression analysis will be used to analyze the relationship between questionnaires’ scoring and both incidence of PU and time to wound healing based on the MNA scores. Predictive validity of both questionnaires for detecting the risk of PU development will be measured through the sensitivity, specificity, and positive and negative predictive values as well as the receiver operating characteristic curve.
2. To analyze the HRQoL of immobilized elderly persons with PUs, acceptability, reliability, and validity as compared to the EuroQol 5D-5L questionnaire will be measured
  - To translate, adapt, and validate the PU-QoL questionnaire from English into Spanish, acceptability, reliability, and validity as compared to the EuroQol 5D-5L questionnaire will be measured.
3. To estimate patients’ nutritional status (MNA-SF), cognitive status (the Reisberg Global Deterioration Scale), and functional independence (the Barthel scale). Mean values and SDs in all three questionnaires will be calculated.
4. To determine the PU-QoL questionnaire’s ability to differentiate between clinical groups (eg, PU severity) and responsiveness to change (eg, wound healing), survival analysis will be performed in order to analyze the relationship between the questionnaire scoring and time to wound healing.

### Ethical Considerations

This project has been approved by the Research Ethics Committee in Human Beings, within the Andalusian Public Health System (Granada) under protocol number PI-0086-2016.

**Table 3:** Database information.

Variable		Description	Coding categories
**Sociodemographic variable**			
	Birthdate	Andalusian Health Service users’ database	dd/mm/yyyy
	Age	From date of birth until date of database entry	Age in years
	Gender	Andalusian Health Service users’ database	Female/male
	Elderly nursing home	Residence	Unique key
**Patient outcomes measures**			
	Braden scale	Braden score questionnaire (individual subscales and total score)	Discrete numeric value (6-23)
	Date of the Braden scale assessment	Date when each repeated Braden questionnaire is completed	dd/mm/yyyy
**Identification variables**			
	NUHSA^a^	Unique identifying electronic health record number within the Andalusian Health Care Service	Unique key
	PHCC^b^	Primary health care center where patient is assisted	Unique key
	Allocation code	Identifies groups of patients allocated to specific health care teams	Unique key
	CNP^c^	Health care practitioners’ number	Unique key
	Registration date	First database record	dd/mm/yyyy
**Follow-up data**			
	Follow-up status	Patient follow-up or status change	Under follow-up, moved away, deceased
	Follow-up status date	Assessment	dd/mm/yyyy
	Type of wound	Only pressure ulcers	Pressure ulcers, other wounds of the skin
	Date of diagnosis	Date wound was first recorded	dd/mm/yyyy
	Date of origin	Date wound was first noticed	dd/mm/yyyy
	Wound stage	Stage of pressure ulcer according to GNEAUPP^d^	0: missing, 1-4: stages I-IV, 5: lesions of deep tissues
	Wound site	Wound location	46 categories
	Associated health care level	Health care level where pressure ulcers appeared	Hospital, nursing home, own home
	Date of wound assessment	Next assessment schedule	dd/mm/yyyy
	Treatment	As prescribed	Text
	Course of treatment	Treatment periodicity	Treatments per time units
	RESVECH^e^ 2.0	Expected assessment results and chronic wounds healing evolution Score	Discrete scale from 0 (already healed) to 35 (worst possible wound)
	Urinary incontinence	Whether incontinence is present at the time of assessment	Yes/no
	Barthel scale	Daily living activities index	Discrete scale from 0 (worst state of disability) to 100 (complete autonomy)
	GDS^f^ scale	GDS	Discrete scale from 1 (no cognitive decline) to 7 (severe dementia)
	MNA-SF^g^ scale	MNA-SF questionnaire	Discrete scale from 0 (worst nutritional state) to 14 (best nutritional state)
	PU-QoL^h^ scale	PU-QoL questionnaire (lower scores are indicative of better results)	10 individual discrete numeric subscales: pain (0-16), exudate (0-16), odor (0-12), sleep (0-12), mobility/movement (0-18), daily activities (0-16), vitality (0-10), emotional well-being (0-30), self-consciousness and appearance (0-14), participation (0-18)
	Mobility status	Patient mobility assessment	Yes/no
	Date of the mobility status assessment	Initial and subsequent mobility assessment record	dd/mm/yyyy

^a^NUHSA: patient health record number.

^b^PHCC: primary health care center.

^c^CNP: health care practitioners’ number.

^d^GNEAUPP: Spanish Advisory Panel on Pressure Ulcers and Chronic Wounds.

^e^RESVECH: wound healing assessment.

^f^GDS: Global Deterioration Scale.

^g^MNA-SF: Mini Nutritional Assessment - Short Form.

^h^PU-QoL: Pressure Ulcer Quality of Life Questionnaire.

## Results

The results of this study are currently under analysis. Ethics approval was provided on March 02, 2017. The start meeting with the ZBS coordinators and DSGM management team was celebrated in June 2017. Throughout September and October 2017, 33 ZBSs across the primary health district agreed to participate in the study and received data collection and registration training in SIRUPP database.

As of April 2019, 362 nurses participated in the study by implementing 11,452 Braden tests, 1608 MNA tests, 960 Barthel tests, and 588 GDS tests. A total of 2983 patients consented to participate, of which 2561 were living at home and 422 were living in nursing houses. In addition, 869 patients developed at least 1 PU since the beginning of the project.

The HRQoL questionnaire translation and cross-cultural adaptation started in November 2017. Its forward translation from English into Spanish ended in February 2018 and continued with backward translation until March 2018. The first Spanish version was evaluated by a group of health professionals and patients between April and May 2018, whose recommendation was to develop a second version of the Spanish HRQoL questionnaire. Starting September 2018, a revised Spanish version was developed and is nowadays implemented with a target population for validation purposes.

The data obtained from the SIRUPP study will provide a detailed outlook on the incidence, mortality, and duration of PUs at local and regional levels. To our knowledge, the validated Spanish-language HRQoL instrument will be the first measure of HRQoL specifically developed for Spanish-speaking patients with PUs.

## Discussion

The SIRUPP project was proposed in view of the burden that PUs exert on the quality and quantity of the life of elderly people. PUs are largely considered to be a preventable condition. Therefore, the SIRUPP project is focused on the characterization of the epidemiology of PUs in our health care system and social setting, with an emphasis on the incidence of PUs and health outcome variability (time needed for the wounds to heal, mortality rates, and HRQoL).

The following challenges were considered in the design of the SIRUPP study: the potential underrepresentation of immobilized patients in the DSGM due to unreported health cases in the SIRUPP data-gathering system and the possible misclassification of patients as immobilized or the misclassification of the PU stage/category.

HRQoL assessment (corresponding to study objective 3) provides valuable, multidimensional knowledge of this major health care problem from the patients’ perspective. The availability of a validated Spanish version of the PU-QoL instrument will be invaluable for optimizing medical decision making for patients with PUs. In addition, this study will allow policymakers to reassess the use and efficacy of evaluation tools currently used in primary health care (ie, the MNA and Braden scales).

## Appendix

Multimedia Appendix 1 Peer-reviewer report from the Progress and Health Foundation (FPS).

## Abbreviations

DSGM: Granada-Metropolitan Primary Healthcare District
GDS: Global Deterioration Scale
GNEAUPP: Spanish Advisory Panel on Pressure Ulcers and Chronic Wounds
HRQoL: health-related quality of life
PU: pressure ulcer
PU-QoL: Pressure Ulcer Quality of Life
SIRUPP: Pressure Ulcer Information and Registration System
SSPA: Andalusian Public Health System
MNA: Mini Nutritional Assessment
MNA-SF: Mini Nutritional Assessment - Short Form
