# Supplementary material for: Monitoring Immobilized Elderly Patients Using a Public Provider Online System for Pressure Ulcer Information and Registration (SIRUPP): Protocol for a Health Care Impact Study
Source: JMIR Res Protoc. 2019 Aug 12;8(8):e13701. doi: 10.2196/13701 (PMC6709896; doi:10.2196/13701)
Supplement: Multimedia Appendix 1 [file resprot_v8i8e13701_app1.pdf]

FINAL ASSESSMENT REPORT
FPS [Progress and Health Foundation] Primary Care Research Projects

Modality	R&I primary care projects
Record	AP-0086-2016
Project title	Analysis of Health Outcomes in Primary Care. Monitoring of Immobilised Elderly Patients through the Pressure Sore Information and Registration System (Sirupp Study)
Main researcher	Vera SALMERÓN EUGENIO
Collaborating researchers	Basilio GÓMEZ POZO, Carmen DOMÍNGUEZ NOGUEIRA, José Antonio EGEA VELÁZQUEZ, José BURGOS SÁNCHEZ, Francisco GONZÁLEZ JIMÉNEZ, Gerardo TIRADO PEDREGOSA, Manuel MONTALVO CABRERIZO, and Claudia RUTHERFORD
Recipient	Fundación para la Investigación Biosanitaria de Andalucía Oriental – Alejandro OTERO [Eastern Andalusian Foundation of Bio-Health Research]
Centre(s)	Granada Metropolitan Primary Care Centre, Zaidín Sur Medical Centre, Churriana de la Vega Medical Centre, University of Sidney
Project score	**78**
Project status (1)	**Financed**

The following are the results of the assessment of the above project by following the criteria set forth under the 7th section of the Permanent Open Call for the execution of research and innovation projects in the field of primary care of the Andalusian Health Service.

	Assessment criteria	Project score	Comparison with the average score of all projects (2)
**Scientific criteria**	1. Scientific-technical quality and feasibility	20/25	+
	2. Research team	6/10	+
	3. Adaptation to budget	4/5	+
	4. Outcome applicability	48/60	+
	**Total scientific assessment score**	**78/100**	**+**
	**TOTAL PROJECT**	**78/100**	**+**

(1) Clarifications on project status

- **Financed**
- **Financed*:** the project has been passed but requires a personal interview to clarify some project-related aspects to eventually obtain funding.
- **Not selected- Improvements must be implemented:** in order to improve the scientific-technical quality of the proposal for future submission of the project to this Call or another, the researchers will receive methodological advice by the EASP [Andalusian School of Public Health], in cooperation with the methodological support service of the corresponding provincial Research Management Foundation. EASP Research Department shall contact the research team to offer them the above support service and specify what aspects of their proposal need to be improved. In case the project is to be submitted again to this primary care Call, it will be reassessed in the next assessment period.
- **Cannot be financed:** upon detection of non-rectifiable concept or methodological errors, the proposal itself will not be financed in any of the assessment periods of this Permanent Open Call. For more information, see the assessment report.

(2) +: Score above average; -: Score below average

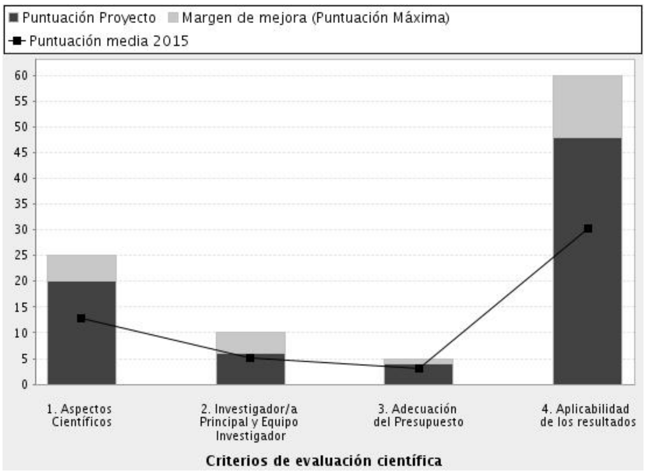

[Translation of the figure, left-right, top-bottom:]

Project score; Room for improvement (top score); 2015 Average score

1. Scientific aspects
2. Main researcher and research team
3. Adaptation to budget
4. Outcome applicability

Scientific assessment criteria

Figure 1. Compared results of scientific project assessment according to each of the criteria.

1. COMMENTS ON SCIENTIFIC ASSESSMENT

This research project is intended to respond to several objectives related to risk factors in the appearance of pressure sores and an estimation of the life quality of patients which have suffered from them.

It would be recommendable to go further into patient selection criteria and the definition of the different study variables, as well as of the monitoring times.
This project is ambitious and pertinent, considering the current evidences of pressure sores in primary care and the implications of such a problem for an increasingly aged and dependent population, as well as for the health system.
A better research team could be formed if more professionals with research project leadership and further publication experience were incorporated.

Criterion 1: Scientific-technical quality and feasibility of the project (20.0/25.0)
The research project is focused on estimating the impact of pressure sores on immobilised elderly patients. The project authors list several goals. There are only a few studies related to the general goal. The designs suggested are basically the monitoring of a prospective cohort and a cross-sectional analysis of the life quality of the patients who have suffered from pressure sores.
A further development of the study might require indicating the monitoring times of each of the cohorts to be selected: the researchers establish that the cohort will be made up of all sore-free patients included in the computer registration system at the beginning of the study. However, it is later specified that patients incorporated into the database as new immobilisation cases throughout the study will also be included in the cohort. Moreover, it would be convenient to refine the criteria used to define the immobilised population: functional criteria, coping, problems of movement?
It would also be recommendable to establish the patient selection methodology for some of the goals, such as the estimation of the predictive values of the scales or the PU-QOL scale validation.
A major potential limitation in the proposal would be the under-registration of immobilised patients in the system and of the different variables to be approached by the researchers.
Some of the variables are not well-defined or used in the shape of acronyms which hinder understanding of what they refer to (NUSHA, CNP, passivity status and date, GDS). It would be important to establish the cause of mortality and/or any underlying pathology behind said mortality.
The researchers explain that the impacts of the pressure sores vary depending on the different units or hospital rooms (associated variables which are not totally reflected in the variables to collect), the variables of which would be necessary for the subsequent statistic analysis suggested.
More clarity should be provided on data sources: it can be inferred that the data registered in the computer system shall be the ones to be used, but then one further data collection system is then referred to, as is the data collection record. In no case does the proposal establish how often the data is to be collected.
The project timeline establishes that field work shall be conducted throughout three years, from the first trimester of 2017 until the third trimester of 2019, which makes it difficult to conduct a final data analysis and its further publication or dissemination.

Criterion 2: Research team. (6.0/10.0)
The main researcher's experience in the approaching of pressure sores is reduced to her current functions in the field of security and in the development of computer systems for registration of the patients who suffer from them. None of her previous publications or research projects addressed such a problem and she does not have any experience in financed project leadership and management. Her experience in research is still rather scarce.
The other members of the research team have little experience in leadership and have participated in only a few financed research projects. One researcher does have the required publications and experience, whose responsibility in the project is, above all, the validation of the scale to assess the quality of life of patients with pressure sores.
The series of tasks and their distribution suggest the need to hire additional staff for the project.

Criterion 3: Adaptation to budget (4.0/5.0)
Some contradictions can be found between the suggested budget for personnel expenditure (22,000 EUR? for the first year, and half the amount for the second and third years) and what has been established under the report (24 months with 3,290 h workload). More clarity should be provided regarding whether partial hiring is required in any of the periods. A reduction can be seen in the Personnel Costs section of the report, given the large number of collaborating researchers in the project.

Criterion 4: Outcome applicability in the Andalusian Public Health System [SSPA] (48.0/60.0)
If the different goals were attained, the outcomes could certainly be applicable to clinical practice and service organisation, as well as published in relevant journals and incorporated into clinical practice guides, or even provide new tools, such as questionnaires. However, the project is not expected to generate outcomes eligible for intellectual property registration.

Remarks:

2. INFORMATION ON KNOWLEDGE PROTECTION
The protection of the knowledge generated in the R&I projects constitutes the basis of the process of assessment and transfer of research outcomes, whose goal is to ensure efficient transmission and application of said outcomes in the health system. Should you require further advice or have any doubts regarding collaborations with companies and/or industrial or intellectual property affairs, do not hesitate to call or email FPS Office of Technological Transfer [FPS-OTT] (Tel.: [+34] 955040450; email address: ott.sspa@juntadeandalucia.es).

The present project is not expected to generate outcomes eligible for registration of intellectual property, other than those derived from previous scientific publications.

3. INFORMATION ON FURTHER FUNDING OPPORTUNITIES AND THE SERVICES PROVIDED BY SSPA NETWORK OF RESEARCH MANAGEMENT FOUNDATIONS (RFGI-SSPA)

For information on **further funding opportunities,** please refer to the following tools which you may find on **INVESTIGA+** R&I Support and Management Services Portal of the SSPA:

- **Call announcement browser:** search the call announcement which best suits the requirements of your activity by type of grant, field, funding entity, status or name. Enter

- **Funding Opportunities Agenda for Biomedical R+D+I,** (A4-printable schedule): planning and browsing tool which includes the basic **requirements** to participate in the calls and a timeline with the effective or expected dates for application submission, as well as a **link to the detailed sheet** of each of the grants. Enter

On **INVESTIGA+,** you may also find all the information available on the range of services offered by the RFGI-SSPA, which provides help, support and legal representation in different locations. It is made up of seven foundations: FCÁDIZ (Cádiz), FIBICO (Córdoba), FABIS (Huelva), FIMABIS (Málaga), FISEVI (Seville), FIBAO (provinces of Granada, Jaén, and Almería) and FPS, as coordinating entity of the Network.

RFGI-SSPA offers researchers the following **range of R+I support services:**

1. Fundraising guidance
- Information on funding opportunities, partner search, business collaboration opportunities (technological demands, public-private collaboration)
- Training in fundraising

2. Grant management
- Preparation and monitoring of proposals
- Economic management of grants and rationale

3. Clinical trial and non-interventional study management
- Information on clinical research opportunities and administrative management of clinical trials
- Guidance on independent clinical research

4. Guidance about international projects
- Preparation of proposals and partner search
- Training/ information

5. Methodological and statistic support
- Search training (SSPA Online Library + OTT-SSPA)
- Methodological advice (projects and PhD theses)
- Statistic analysis and database design

6. Outcome protection and transfer guidance and management
- Guidance about publications and translation services
- Public-private cooperation agreements
- Outcome protection and transfer guidance and management
